# Death from Chloroform

**Published:** 1858-12

**Authors:** 


DEATH FROM CHLOROFORM.

Dr. Robert Lee, of London, reports, in the *Med. Times and Gazette*, a case of death from chloroform, occurring in the practice of Dr. John Campbell, of Largs, in Scotland. The presentation was natural. The woman had taken chloroform in six previous labors without accident. The pains becoming active, she called for chloroform, and inhaled about ʒij., "threw herself violently back, gave a gasp or two, a slight gurgle was heard, and respiration and the pulse instantly ceased." Dr. Campbell arrived a few minutes after the death. Having no instrument, he did not deliver the child. The head rested on the perineum. It is only a few weeks since it was publicly denied that any case of death from chloroform, during labor, had ever occurred in Scotland.

